# Tunable Non-Enzymatic Glucose Electrochemical Sensing Based on the Ni/Co Bimetallic MOFs

**DOI:** 10.3390/molecules28155649

**Published:** 2023-07-26

**Authors:** Qi Wang, Qi Jia, Peng Hu, Liudi Ji

**Affiliations:** 1 School of Pharmacy, Hubei University of Science and Technology, Xianning 437100, China; hy33601@163.com (Q.W.); jq_958@163.com (Q.J.); 2 Hubei Key Laboratory of Radiation Chemistry and Functional Materials, Hubei University of Science and Technology, Xianning 437100, China

**Keywords:** 2D MOFs, metal centers, ligands, tunable electrochemistry, glucose sensing

## Abstract

Constructing high-performance glucose sensors is of great significance for the prevention and diagnosis of diabetes, and the key is to develop new sensitive materials. In this paper, a series of Ni_2_Co_1_-L MOFs (L = H_2_BPDC: 4,4′-biphenyldicarboxylic acid; H_2_NDC: 2,6-naphthalenedicarboxylic acid; H_2_BDC: 1,4-benzenedicarboxylic acid) were synthesized by a room temperature stirring method. The effects of metal centers and ligands on the structure, compositions, electrochemical properties of the obtained Ni_2_Co_1_-L MOFs were characterized, indicating the successful preparation of layered MOFs with different sizes, stacking degrees, electrochemical active areas, numbers of exposed active sites, and glucose catalytic activity. Among them, Ni_2_Co_1_-BDC exhibits a relatively thin and homogeneous plate-like morphology, and the Ni_2_Co_1_-BDC modified glassy carbon electrode (Ni_2_Co_1_-BDC/GCE) has the highest electrochemical performance. Furthermore, the mechanism of the enhanced glucose oxidation signal was investigated. It was shown that glucose has a higher electron transfer capacity and a larger apparent catalytic rate constant on the Ni_2_Co_1_-BDC/GCE surface. Therefore, tunable non-enzymatic glucose electrochemical sensing was carried out by regulating the metal centers and ligands. As a result, a high-sensitivity enzyme-free glucose sensing platform was successfully constructed based on the Ni_2_Co_1_-BDC/GCE, which has a wide linear range of 0.5–2899.5 μM, a low detection limit of 0.29 μM (S/N = 3), and a high sensitivity of 3925.3 μA mM^−1^ cm^−2^. Much more importantly, it was also successfully applied to the determination of glucose in human serum with satisfactory results, demonstrating its potential for glucose detection in real samples.

## 1. Introduction

Glucose is one of the essential nutrients for human metabolism, but diabetes can be triggered when blood sugar levels are higher than normal. As we all know, diabetes is one of the most common chronic diseases in our daily life has and is a serious threat to people’s health [1,2]. Therefore, there is a significant medical value and commercial prospect to developing a stable and accurate glucose sensor for rapid real-time monitoring of blood glucose levels. To develop cheap and practical sensors, researchers have tried many new materials, according to the different catalytic mechanisms of these materials. The detection methods can be divided into colorimetry, fluorescence spectrometry, electrochemical method, etc. [3,4,5]. In contrast, the electrochemical method has the advantages of being convenient, rapid, sensitive, and economical and is the most widely used method for the determination of glucose content [6,7]. At present, most of the small blood glucose monitors on the market are enzyme-based blood glucose sensors, which have the advantages of high sensitivity and selectivity. However, there are also some insurmountable defects, such as the high cost and harsh survival conditions of biomolecular enzymes, and they are also susceptible to the influence of the external environment, such as temperature and pH. Therefore, it is essential to prepare enzyme-free glucose sensors that have a simple in process, are less influenced by external factors, and are easily stored. Recently, enzyme-free glucose sensors have been developed by researchers using precious metals [8,9,10], inorganic carbon materials [11,12], transition metals and their oxides [13,14,15], nitrides and phosphides [16,17]. However, many of them still suffer from low catalytic activity, low sensitivity, and surface poisoning. Therefore, it is crucial to develop new high-performance, highly sensitive, and selective nanomaterials to construct efficient glucose sensors.

Metal-organic frameworks (MOFs) are a kind of porous coordination polymer with periodic network structures that are composed of metal ions or metal clusters and organic ligands [18]. With the advantages of an abundant ligand source and metal source, orderly and adjustable porous structure, large specific surface area, good thermal stability, they have been widely used in gas storage and separation [19,20], energy storage and conversion [21,22], molecular detection and contaminant identification [23,24], etc. Moreover, MOFs are rich in unsaturated metal active sites, which gives them good potential for electrochemical catalysis and sensing [25,26]. Compared with conventional bulk MOFs, 2D MOFs have nanoscale thickness and more uniform dimensions, which are favorable for mass transfer and fast electron transfer. Furthermore, the lamellar structure can expose more accessible active sites, which results in better electrochemical properties [27,28,29]. In addition, it was shown that there is a synergistic effect between the metals of bimetallic MOFs, which can effectively improve the catalytic activity [30,31]. Therefore, the construction of a highly sensitive and selective glucose electrochemical monitoring platform with 2D bimetallic MOFs has a very promising future. Moreover, it is innovative to carry out the research on how the metal centers and ligands affect glucose sensing performance.

Nickel and cobalt are widely used in the field of glucose catalysis because of their abundant source, low price, and high electrocatalytic activity [32,33]. So, in this study, Ni and Co were selected as metal centers, dicarboxylic acids of different chain lengths (H_2_BPDC, H_2_NDC, H_2_BDC) were chosen as organic ligands, and a series of Ni_2_Co_1_-L MOFs (Ni_2_Co_1_-BDC, Ni_2_Co_1_-NDC, Ni_2_Co_1_-BPDC) were prepared by the room temperature stirring method. The patterns of influence of metal center types, ratios, ligands on their electrochemical properties were investigated. The Ni_2_Co_1_-BDC modified glassy carbon electrode (Ni_2_Co_1_-BDC/GCE) has a relatively large electrochemically active area and exposes more accessible catalytically active sites with high glucose catalytic activity. The mechanism of glucose oxidation signal enhancement was further investigated. The results demonstrated that glucose has a high electron transfer capacity and a large apparent catalytic rate constant on the Ni_2_Co_1_-BDC/GCE surface. Ultimately, a highly sensitive enzyme-free glucose sensing platform was successfully constructed based on the Ni_2_Co_1_-BDC/GCE. It was used for an actual human serum assay, and the results were consistent with hospital assays, indicating its good application prospects.

## 2. Results and Discussion

### 2.1. Characterization of Ni_2_Co_1_-L MOFs

**Figure 1:**
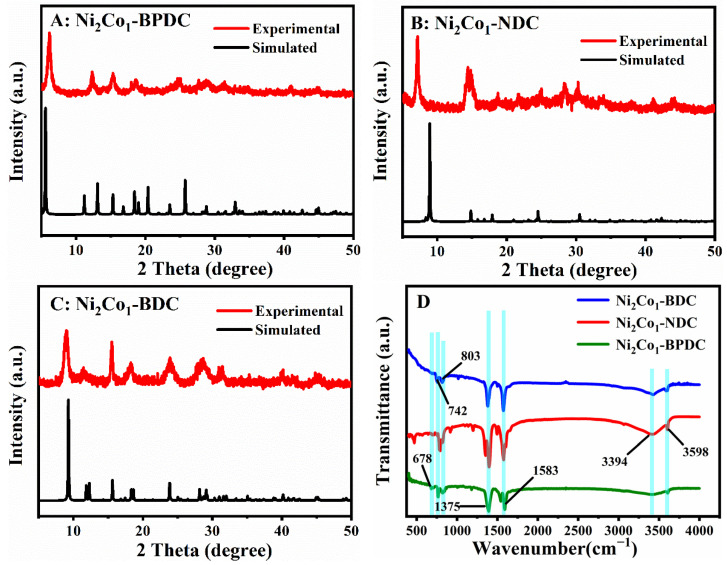
(**A**–**C**) XRD patterns of Ni_2_Co_1_-L MOFs. (**D**) FTIR spectra of Ni_2_Co_1_-L MOFs.

Additionally, the crystallinity and phase purity of the synthesized Ni_2_Co_1_-L MOFs were examined using XRD, and the results are shown in Figure 1A–C. With the exception of some deviations in the relative intensities, their XRD patterns were in good agreement with the simulated patterns, which indicated the formation of high purity [34,35,36]. By comparing the FTIR spectra in Figure 1D, it can be seen that some absorption bands are the same in Ni_2_Co_1_-BPDC, Ni_2_Co_1_-NDC, and Ni_2_Co_1_-BDC MOFs. These indicated that they share common organic functional groups. The bending vibrations of the aromatic ring are about 678, 742, and 803 cm^−1^ [37]. Furthermore, they all have two characteristic peaks around 1375 and 1583 cm^−1^, which correspond to the asymmetric stretching vibration peak (*as*) and the symmetric (*ss*) stretching vibration peak of the carboxyl group. The presence of a broad absorption band at 3394 cm^−1^ and the characteristic peak at 3598 cm^−1^ are due to the -OH vibration of water molecules present in the material structures [38]. All of these confirmed the successful introduction of ligands into the synthesized MOFs. In particular, the XRD diffraction peaks and FTIR patterns of Ni_2_Co_1_-BDC were basically in agreement with those of the monometallic Ni-BDC and Co-BDC (Figure S1A,B in Supporting Information), which indicated that the introduction of Ni, Co bimetals did not affect the crystalline structure of MOFs. TGA analysis was applied to investigate the thermal stability of Ni_2_Co_1_-L MOFs in the range of 25–700 °C. The results are shown in Figure S1C. The main weight loss of Ni_2_Co_1_-L MOFs in the range of 25–170 °C was attributed to water and residual solvents adsorbed within the pores [39]. The second weight loss around 200–280 °C was attributed to coordinated water molecules, and the weight loss around 400 °C was assignable to decomposition of the carboxylate ligand [40]. As a comparison, Ni_2_Co_1_-BDC had less percent weight loss, which resulted in better stability.

**Figure 2:**
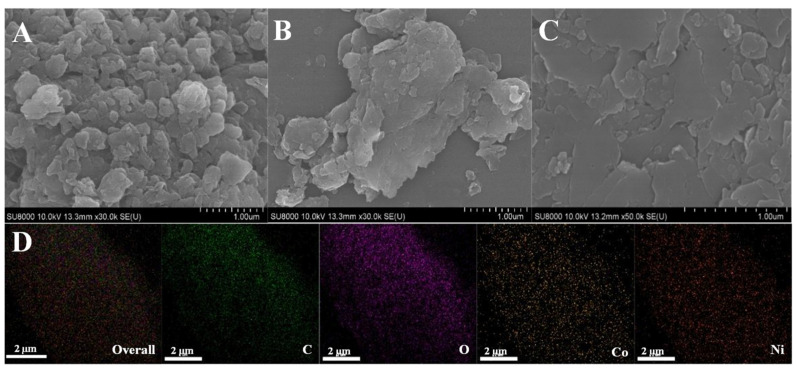
SEM images of Ni_2_Co_1_-BPDC (**A**), Ni_2_Co_1_-NDC (**B**), Ni_2_Co_1_-BDC (**C**). (**D**) EDS mapping images of Ni_2_Co_1_-BDC.

Then, the morphological characteristics of the synthetic materials were investigated using the SEM technique, and the corresponding images are shown in Figure 2**.** It can be seen from Figure 2A that the Ni_2_Co_1_-BPDC is a layered stacking structure consisting of nanosheets with smaller sizes. Ni_2_Co_1_-NDC (Figure 2B) is a bulky structure. On the contrary, Ni_2_Co_1_-BDC (Figure 2C) is a relatively thin and homogeneous plate-like morphology, which possesses a 2D structure at the micron size. Then, the elemental distribution and content were characterized by EDS. From Figure 2D and Table S1 (Supporting Information), it can be seen that C, O, Ni, and Co are uniformly distributed on the surface of Ni_2_Co_1_-BDC.

### 2.2. Electrochemical Performance of Ni_2_Co_1_-L MOFs

**Figure 3:**
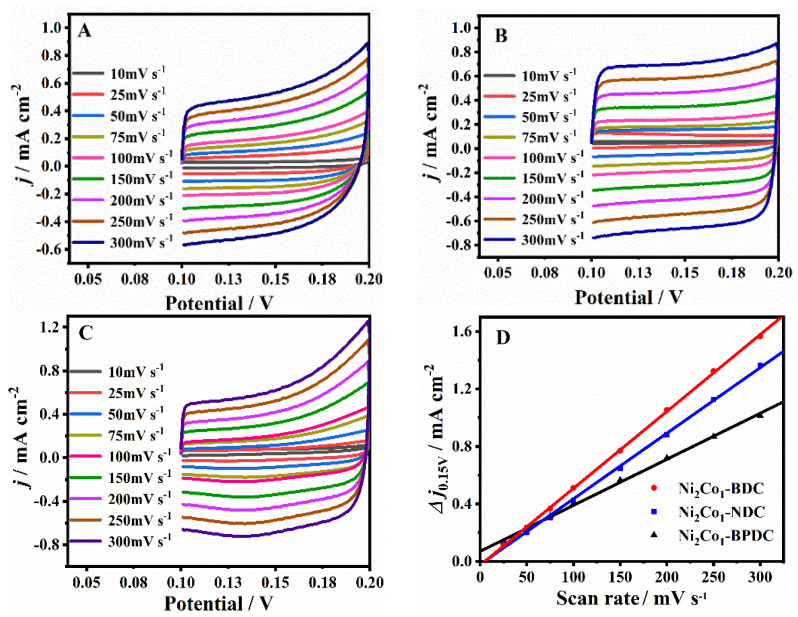
CV curves of the Ni_2_Co_1_-BPDC/GCE (**A**), Ni_2_Co_1_-NDC/GCE (**B**), Ni_2_Co_1_-BDC/GCE (**C**) at different scan rates in 1 M KOH; (**D**) linear plot of current density versus scan rates at 0.15 V.

First of all, the electrochemical active areas of different Ni_2_Co_1_-L MOF nanosheets were compared by calculating the electric double-layer capacitance (*C_dl_*) based on the CV tests in a non-Faradaic region (e.g., from 0.10 to 0.20 V) [41]. The results are shown in Figure 3A–C. According to the slope of the linear relationship between the capacitive currents difference (Δ*j* = *j_anodic_* − *j_cathodic_*) at 0.15 V and the scan rates (Figure 3D), the *C_dl_* of the Ni_2_Co_1_-BPDC/GCE, Ni_2_Co_1_-NDC/GCE, and Ni_2_Co_1_-BDC/GCE was obtained to be 3.20, 4.65, and 5.35 mF cm^−2^, respectively. It is known that the *C_dl_* is proportional to the electrochemically active area. The Ni_2_Co_1_-BDC/GCE had the largest active area, which may be derived from its 2D lamellar morphology.

**Figure 4:**
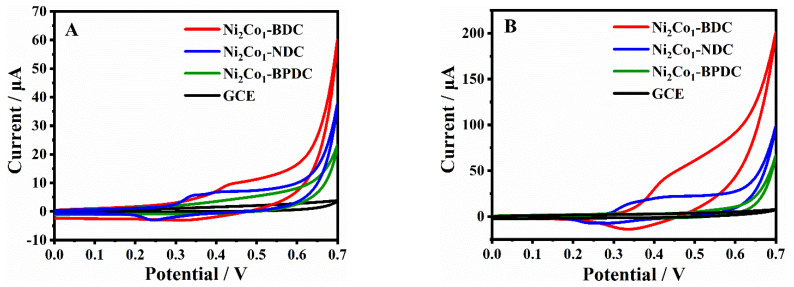
(**A**) CV curves for GCEs at 100 mV/s in 0.1 M NaOH. (**B**) CV curves for GCEs at 100 mV/s in 0.1 M NaOH with 0.1 mM glucose.

It is well known that one of the key factors affecting the catalytic performance of electrode material is the number of active sites, which can be measured in the electrochemically active region. Here, the electrocatalytic activity of Ni_2_Co_1_-L MOFs was compared by recording the CV curves in 0.1 M NaOH at a scan rate of 100 mV/s, and the results are shown in Figure 4A. It is noteworthy that the GCE and Ni_2_Co_1_-BPDC/GCE show no obvious redox peak, suggesting poor electrochemical activity. The Ni_2_Co_1_-NDC/GCE shows a pair of asymmetric redox peaks at around 0.42 and 0.24 V, and in the CV curves of the Ni_2_Co_1_-BDC/GCE more obvious redox peaks appear at around 0.46 and 0.34 V. These redox peaks most likely resulted from the redox of their metal centers Ni^2+^ and Co^2+^. Additionally, the Ni_2_Co_1_-BDC/GCE has a higher peak current, suggesting the largest number of exposed active sites. Then, glucose was chosen as the target molecule and the catalytic activity of these materials on glucose was investigated. Figure 4B shows the CV curves of different GCEs after the addition of 0.1 mM glucose. No obvious redox peaks were observed on the surfaces of the GCE and Ni_2_Co_1_-BPDC/GCE. For the Ni_2_Co_1_-NDC/GCE and Ni_2_Co_1_-BDC/GCE, both of them show an oxidation peak with enhanced signal compared with Figure 4A without glucose. It was found that the Ni_2_Co_1_-BDC/GCE had the most obvious phenomenon, which indicated that it had the strongest catalytic activity for the oxidation of glucose. This can be attributed to the unique disk-like structure of Ni_2_Co_1_-BDC, as it exposes more active sites easily, like the results we mentioned above. The electrode reaction mechanism of the redox process can be expressed by the following equation [42]:

Ni^2+^-H_2_BDC → Ni^3+^-H_2_BDC + e^−^ (1)

Ni^3+^-H_2_BDC + OH^−^ + glucose → Ni^2+^-H_2_BDC glucolactone + H_2_O + e^−^ (2)

Co^2+^-H_2_BDC → Co^3+^-H_2_BDC + e^−^ (3)

Co^3+^-H_2_BDC + OH^−^ + glucose → Co^2+^-H_2_BDC glucolactone + H_2_O + e^−^ (4)

### 2.3. Electrochemical Oxidation of Glucose on Ni_2_Co_1_-L/GCEs

**Figure 5:**
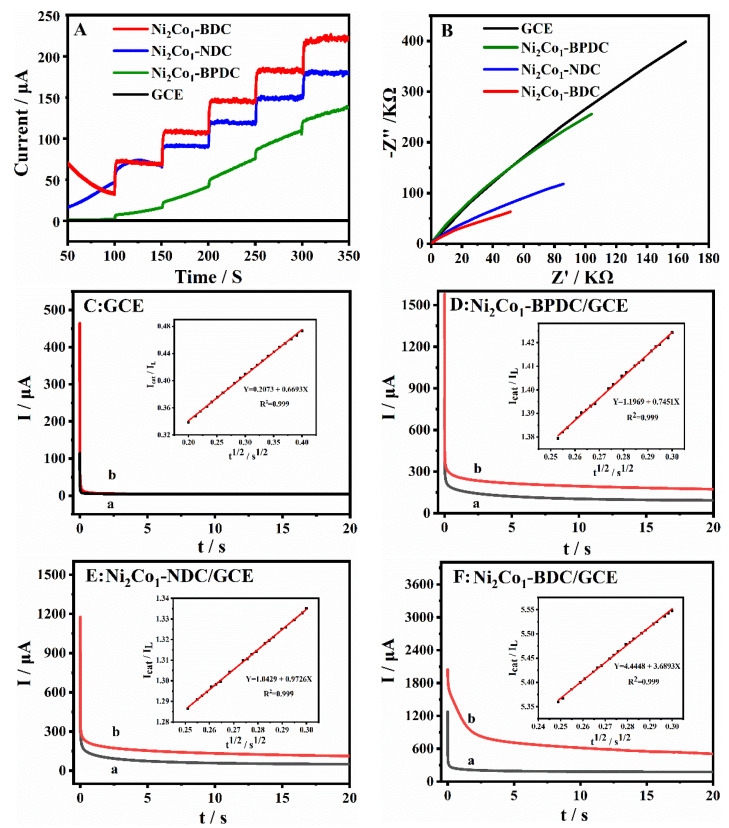
(**A**) *i-t* curves of different GCEs at the continuous injection of 0.1 mM glucose. (**B**) Nyquist curves of different GCEs in 0.1 M NaOH containing 0.1 mM glucose. Frequency range: 0.1 Hz–100 kHz. (**C**–**F**) Chronoamperometry behaviors on different GCEs in the absence (a) or presence of 1 mM glucose in 0.1 M NaOH (b), inset: plots of *I*_cat_/*I*_L_-*t* ^1/2^.

To further demonstrate the electrocatalytic activity of the series Ni_2_Co_1_-L MOFs for glucose oxidation, the *i-t* response of glucose on the surface of different GCEs was investigated. As shown in Figure 5A, *i-t* curves were recorded at 0.5 V for different GCEs after successive addition of 0.1 mM glucose in 0.1 M NaOH solution. No current step signal of glucose was observed on the bare GCE, indicating poor electrochemical activity. The current step signal of the Ni_2_Co_1_-BPDC/GCE had a slight increase, but it is difficult to maintain a smooth state. In contrast, a remarkable increase was noticed in both the Ni_2_Co_1_-NDC/GCE and Ni_2_Co_1_-BDC/GCE, and the current step signal of the Ni_2_Co_1_-BDC/GCE had the largest value.

To explore the reason for the signal enhancement effects for the oxidation of glucose, electrochemical impedance spectroscopy (EIS) measurements were employed to investigate the electron transfer resistance of different Ni_2_Co_1_-L/GCEs during glucose oxidation. In the Nyquist plot, the semi-circle diameter represents the charge transfer resistance (*R_ct_*) for the electrode surface active species [43]. Herein, based on Figure 5B, the fitted values of *R_ct_* for the oxidation of glucose on the GCE, Ni_2_Co_1_-BPDC/GCE, Ni_2_Co_1_-NDC/GCE, and Ni_2_Co_1_-BDC/GCE were 85.4, 43.3, 27.2, and 12.0 KΩ, respectively. The greatly decreased *R_ct_* values revealed that the Ni_2_Co_1_-BDC/GCE improved the electron transfer ability of glucose, consequently resulting in higher oxidation signals.

Subsequently, the chronoamperometry experiment was applied to study the electrochemical kinetics property, which can explain the signal enhancement mechanism more deeply. Figure 5C–F shows the charge (*Q*)-time (*t*) curves for the GCE, Ni_2_Co_1_-BPDC/GCE, Ni_2_Co_1_-NDC/GCE, and Ni_2_Co_1_-BDC/GCE in 0.1 M NaOH in the absence (a) and presence of 1 mM glucose (b). The apparent catalytic rate constant (*K_cat_*) was determined according to the equation [44].

*I*_cat_/*I*_L_ = (π*k*_cat_*Ct*)^1/2^ (5)

where *I*_cat_ represents the catalytic current of analytes on the Ni_2_Co_1_-L/GCEs; *I*_L_ is the limiting current in the absence of analytes; *C* and *t* represent the analyte’s concentration and time elapse, respectively. The linear regression equation between *I*_cat_/*I*_L_ with *t*^1/2^ is given in the inset of Figure 5C–F. The *k*_cat_ for the GCE, Ni_2_Co_1_-BPDC/GCE, Ni_2_Co_1_-NDC/GCE, and Ni_2_Co_1_-BDC/GCE was calculated as 1.43 × 10^2^, 1.77 × 10^2^, 3.01 × 10^2^, and 3.69 × 10^3^ M^−1^·s^−1^. In summary, the Ni_2_Co_1_-BDC/GCE had the strongest electron transfer capacity and the highest electrocatalytic rate constant for glucose oxidation and consequently exhibited the largest signal response. The possible reasons are summarized as follows: First, the Ni, Co bimetallic provided active metal oxidation sites for glucose. Second, the 2D lamellar structure was more beneficial to the contact of active sites and accelerated electron transfer and mass transfer. In summary, the Ni_2_Co_1_-BDC/GCE had good electrochemical properties, which can be applied to the construction of a glucose electrochemical sensing platform.

**Figure 6:**
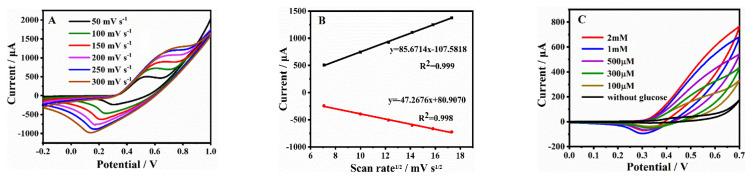
(**A**) CV curves of the Ni_2_Co_1_-BDC/GCE in the presence of 0.1 mM glucose at different scan rates from 50 to 300 mV/s in 0.1 M NaOH. (**B**) The plots of peak cathode and anode currents versus the square root of the scan rate. (**C**) CV curves of the Ni_2_Co_1_-BDC/GCE in 0.1 M NaOH in response to different glucose concentrations.

To further investigate the reaction kinetic characteristics of the Ni_2_Co_1_-BDC/GCE for glucose oxidation, the CV curves of the electrode at different sweep rates (50–300 mV/s) were tested in the NaOH solution containing 0.1 mM glucose (Figure 6A). It can be seen from Figure 6B that both anodic and cathodic peak currents (*I_p_*) increased in proportion to the square root of the scan rates. These results indicated that the electrochemical kinetics were diffusion-controlled. Moreover, the anodic peak potentials (*E_pa_*) shifted more positively and the cathodic potentials (*E_pc_*) shifted more negatively with increases in scan rates, which suggested the quasi-reversible electrochemical reaction process of the Ni_2_Co_1_-BDC on the electrode surface in the majority of activation sites [45]. Figure 6C presents the CVs of the Ni_2_Co_1_-BDC/GCE, including various concentrations of glucose. It was found that the peak current density of anodic oxidation increased with the augment of glucose concentration from 0 to 2 mM, which indicated that the Ni_2_Co_1_-BDC/GCE had good catalytic performance for glucose oxidation.

### 2.4. Non-Enzymatic Glucose Sensing Based on the Ni_2_Co_1_-BDC/GCE

**Figure 7:**
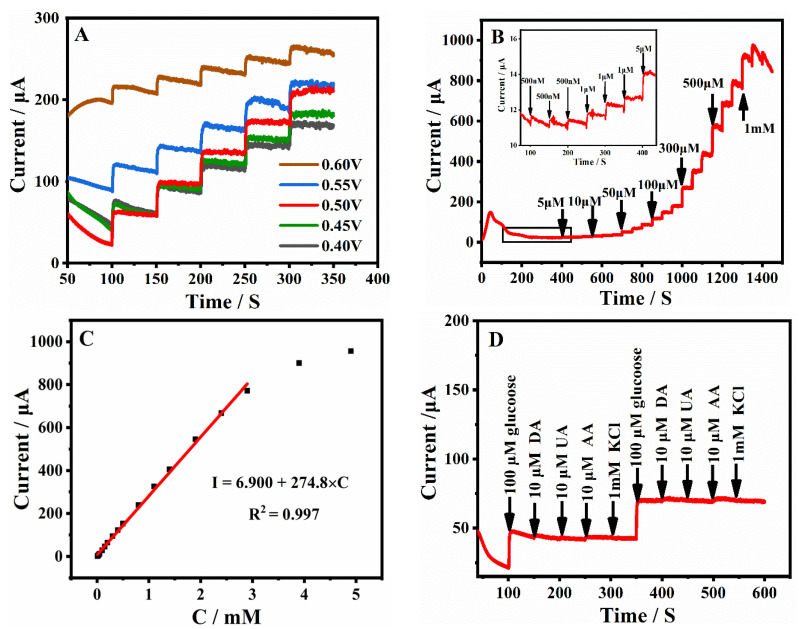
(**A**) *i-t* curves of Ni_2_Co_1_-BDC at different successive injections of 0.1 mM glucose. (**B**) Amperometric response of Ni_2_Co_1_-BDC to different concentrations of glucose at 0.50 V. (**C**) Linear function of amperometric response and glucose concentration. (**D**) Amperometric response of injected glucose (0.1 mM) and a series of interfering compounds at 0.50 V.

The Ni_2_Co_1_-BDC/GCE was used to construct the enzyme-free glucose sensing because of its excellent electrochemical properties. To achieve the optimum sensing performance, the material preparation conditions, such as the effects of the bimetallic type and ratio (Ni, Ni:Co = 1:1, 2:1, 1:2, Co), the type and concentration of the applied base source (NaOH, KOH, TEA, TEAH) on the glucose oxidation signal were optimized. As shown in Figure S2A–C, the strongest current step signal was obtained with the Ni_2_Co_1_-BDC/GCE (3 mmol KOH). Then, the test conditions (potential, modification amount, dispersing solvent) were also optimized. As shown in Figure 7A and Figure S2D–F, the strongest current step signal was obtained under the test conditions of the test voltage of 0.50 V, dispersing solvent of DMF, and modification amount of 4 μL (5 μL Nafion).

The relationship between glucose concentration and its oxidation signal was investigated under optimal conditions. Figure 7B shows the amperometric responses obtained for the Ni_2_Co_1_-BDC/GCE with successive additions of different concentrations of glucose in 0.1 M NaOH solution under the above optimal conditions. An excellent linear relationship was observed between the glucose concentration and its response signal in the range of 0.5 to 2899.5 μM (Figure 7C). The linear regression equation was described as *I*_pa_ (μA) = 274.8 *C* (mM) + 6.900 with R^2^ = 0.997. The sensitivity of the Ni_2_Co_1_-BDC/GCE was 3925.3 μA mM^−1^ cm^−2^, and the detection limit was 0.29 μΜ (S/N = 3). The current response was gradually saturated with the gradual increase in glucose concentration, probably because the electrode surface was partially covered by adsorbed reaction intermediates, thus not providing enough electroactive sites for the oxidation process of glucose [46]. The Ni_2_Co_1_-BDC/GCE constructed in this study had a higher sensitivity, a lower detection limit, and a wider linearity range than the other transition metal electrochemical sensors constructed with enzyme-free glucose previously reported in the literature, as summarized in Table 1. Therefore, the Ni_2_Co_1_-BDC/GCE was found to have broad application prospects in the construction of glucose sensors.

The interference of some possible co-existing biomolecules with the detection of glucose signals at Ni_2_Co_1_-BDC/GCE electrodes was investigated. The concentration of glucose in human serum is considered to be about 10 times higher than the concentration of the interfering biomolecule in the anti-interference test [47]. Therefore, the anti-interference test of the Ni_2_Co_1_-BDC/GCE was performed by successive addition of 100 μM glucose, 10 μM ascorbic acid (AA), 10 μM dopamine (DA), 10 μM uric acid (UA), and 1 mM KCl in 0.1 M NaOH. As can be seen from Figure 7D, the Ni_2_Co_1_-BDC/GCE had a very obvious current response to glucose, but the current response to these interferences was almost negligible, indicating that the sensor had good anti-interference ability.

**Table 1:** Performance comparison with previously reported glucose sensors.

Modified Electrode	Linear Range (µM)	LOD (µM)	Sensitivity (µA mM^−1^cm^−2^)	Ref.
Ni/ZAC-O NTAs	0–2800	1.02	1553	[48]
NiCo-hmf	1000–10,000	0.31	1739.04	[49]
NiO nanofiber/GO	2–600	0.8	1100	[50]
Mn_3_O_4_/N-doped rGO	2.5–529.5	1	1423.9	[51]
Ni@Cu-MOF	5–2500	1.67	1703.33	[52]
Ni_2_Co_1_-BDC/GCE	0.5–2899.5	0.29	3925.3	This work

Ni/ZAC-O NTAs: Ni-decorated ZrAlCo-O nanotube arrays; hmf: hydroxide microflowers.

Moreover, the reproducibility of the proposed Ni_2_Co_1_-BDC/GCE-based sensors was further evaluated. The relative standard deviation (RSD) of five independently manufactured Ni_2_Co_1_-BDC/GCE measurements of 100 μM glucose was calculated as 3.06%. Meanwhile, the RSD of five consecutive glucose additions with one Ni_2_Co_1_-BDC/GCE was only 2.14%. The lower RSD values justified the good reproducibility of the fabricated sensors. Several Ni_2_Co_1_-BDC/GCEs were stored in air at room temperature, and the current responses for 100 μM glucose were measured each day. The current response retained 93.37% of its original value after a week, as illustrated in Figure S2G, indicating good stability of the as-prepared electrochemical sensor.

To study the practical value of the Ni_2_Co_1_-BDC/GCE, the *i-t* method was used to detect human serum samples, and the accuracy and reliability of the electrode were evaluated. The standard glucose solution and different amounts of serum were added sequentially to 10 mL of 0.1 M NaOH at a voltage of 0.50 V (Figure S2H, Supporting Information). Then, blood glucose data measured from different human serum samples were compared with hospital tests, which showed satisfying results (Table 2). It can be seen that the blood glucose concentrations obtained from the Ni_2_Co_1_-BDC/GCE were well consistent with the hospital test results, which demonstrated the reliable practical value of the developed glucose sensor.

## 3. Materials and Methods

### 3.1. Reagents and Solutions

Co(CH_3_COO)_2_·4H_2_O, Ni(CH_3_COO)_2_·4H_2_O, 4,4′-biphenyldicarboxylic acid (H_2_BPDC), 2,6-naphthalenedicarboxylic acid (H_2_NDC), 1,4-benzenedicarboxylic acid (H_2_BDC), KOH, NaOH, KCl, ascorbic acid (AA), uric acid (UA), dopamine (DA), glucose, Ethanol, and N,N-Dimethylformamide (DMF) were bought from Sinopharm Chemical Reagent (Shanghai, China). Pure water (15 MΩ) used in the experiments was supplied by a Millipore System (Milli Q). Human serum was provided by the Second Affiliated Hospital of Hubei University of Science and Technology.

### 3.2. Instruments

Electrochemical performance experiments were carried out using a CHI 660E electrochemical workstation with a traditional three-electrode system. The saturated calomel electrode (SCE, Rosemead, CA, USA, in saturated KCl solution), platinum wire electrode, and the modified glassy carbon electrode (GCE) served as the reference electrode, counter electrode and the working electrode, respectively. The morphological characterization of the Ni_2_Co_1_-L MOFs was performed with a scanning electron microscope (SEM, Hitachi SU-8000) operated at an accelerating voltage of 10 kV. The composition and crystal structure of the Ni_2_Co_1_-L MOFs were checked by X-ray diffraction (XRD, Rigaku RINT 2500×) with Cu-Ka radiation. The Fourier transform infrared (FTIR) spectra of the samples were collected using the Avatar 360 Nicolet instrument.

### 3.3. Synthesis of Ni_2_Co_1_-L MOFs

In a typical preparation procedure, 0.67 mmol (0.1667 g) Ni(CH_3_COO)_2_·4H_2_O and 0.33 mmol (0.0822 g) Co(CH_3_COO)_2_·4H_2_O (Ni:Co = 2:1) were dissolved in 5 mL of DMF to form solution A. Meanwhile, 5 mL of ultrapure water was used to dissolve 3 mmol (0.1680 g) of KOH, and then 1 mmol of organic ligand (L = H_2_BPDC, 0.2423 g; H_2_NDC, 0.2162 g; H_2_BDC, 0.1663 g) was added. After the above solution was clear, 5 mL of DMF was added to form solution B. Solution A was poured into solution B and reacted for 1 h under rapid stirring conditions. Finally, the mixed solution was centrifuged at 6000 rpm for 10 min. The obtained precipitate was centrifuged and washed by DMF and ethanol (1:1) several times and dried at 60 °C. Finally, Ni_2_Co_1_-BPDC, Ni_2_Co_1_-NDC, and Ni_2_Co_1_-BDC MOFs were obtained.

### 3.4. Preparation of the Ni_2_Co_1_-L MOFs Modified GCE

For the modification, 5 mg/mL of the material suspension was prepared by ultrasonic dispersion of 2.5 mg of the Ni_2_Co_1_-L MOFs powder in 495 μL of DMF, followed by the addition of 5 μL of Nafion. Meanwhile, the surface of the GCE (diameter: 3.0 mm) was polished using 0.05 µm alumina slurry and then washed with ethanol and ultrapure water in an ultrasonic bath. After that, 4 μL of Ni_2_Co_1_-L dispersion was pipetted onto the surface of the GCE, which was dried with an infrared baking lamp. Then, the Ni_2_Co_1_-BPDC/GCE, Ni_2_Co_1_-NDC/GCE, Ni_2_Co_1_-BDC/GCE were obtained and used as the working electrodes.

## 4. Conclusions

In summary, a series of Ni/Co bimetallic MOFs (Ni_2_Co_1_-BPDC, Ni_2_Co_1_-NDC, Ni_2_Co_1_-BDC) were successfully prepared under simple room temperature conditions. The effects of metal centers and ligand types on the morphology and electrochemical sensing properties of the Ni_2_Co_1_-L MOFs were demonstrated by different characterization approaches. It was demonstrated that Ni_2_Co_1_-BDC had an ultrathin and a homogeneous disk-like structure with a large electrochemical active area, which can expose abundant active sites. Moreover, an excellent electrochemical sensing capability for glucose detection was demonstrated by the prepared electrochemical sensor, which obtained a wider linear range, a lower detection limit, and a more stable interference immunity. Last, satisfactory results were also found for the detection of human serum samples, and the utility and accuracy were further demonstrated.

## Figures and Tables

**Table 2:** Detection of glucose in human serum samples by the Ni_2_Co_1_-BDC/GCE and glucometer.

No.	by Glucometer (mM)	Ni_2_Co_1_-BDC/GCE (mM)	Relative Error
1	5.61	5.70	1.6%
2	6.04	6.05	0.2%
3	5.76	5.56	−3.5%
4	4.95	4.92	−0.61%

## Data Availability

Data will be made available upon reasonable request.

## Supplementary Materials

The following supporting information can be downloaded at: https://www.mdpi.com/article/10.3390/molecules28155649/s1, Figure S1: (**A**) XRD and (**B**) FTIR spectra of monometallic and bimetallic MOF; (**C**) thermal gravimetric analysis (TGA) curve of Ni_2_Co_1_-L MOFs. Figure S2. *i-t* curves of MOFs under different experimental conditions with the continuous injection of 0.1 mM glucose. (**A**) metal center and scale; (**B**) types of alkali; (**C**) amounts of KOH; (**D**) the volume of Nafion; (**E**) modified volume; (**F**) dispersing solvents; (**G**) stability of the Ni_2_Co_1_-BDC/GCE in 0.1 M NaOH solution containing 0.1 mM Glu; (**H**) amperometric response of the Ni_2_Co_1_-BDC/GCE at successive additions of 20 μM glucose, 20 μL serum, and 40 μL serum, followed by 20 μM glucose. Table S1. The element content of Ni_2_Co_1_-BDC.

## Sample Availability

Samples of the compounds are available from the authors upon reasonable request.
