# Supplementary material for: Tunable Non-Enzymatic Glucose Electrochemical Sensing Based on the Ni/Co Bimetallic MOFs
Source: Molecules. 2023 Jul 26;28(15):5649. doi: 10.3390/molecules28155649 (PMC10420269; doi:10.3390/molecules28155649)
Supplement: Supplementary file 1 [file molecules-28-05649-s001.zip › molecules-2507200-supplementary.pdf]

Supporting Information

Tunable non-enzymatic glucose electrochemical sensing based on the Ni/Co bimetallic MOFs

Qi Wang [1,2], Qi Jia [1,2], Peng Hu [2,*] and Liudi Ji [2,*]

[1] School of Pharmacy, Hubei University of Science and Technology, Xianning 437100, China
[2] Hubei Key Laboratory of Radiation Chemistry and Functional Materials, Hubei University of Science and Technology, Xianning 437100, China
* Correspondence: jiliudi@126.com (L.J.); hupeng@hbust.edu.cn (P. Hu).

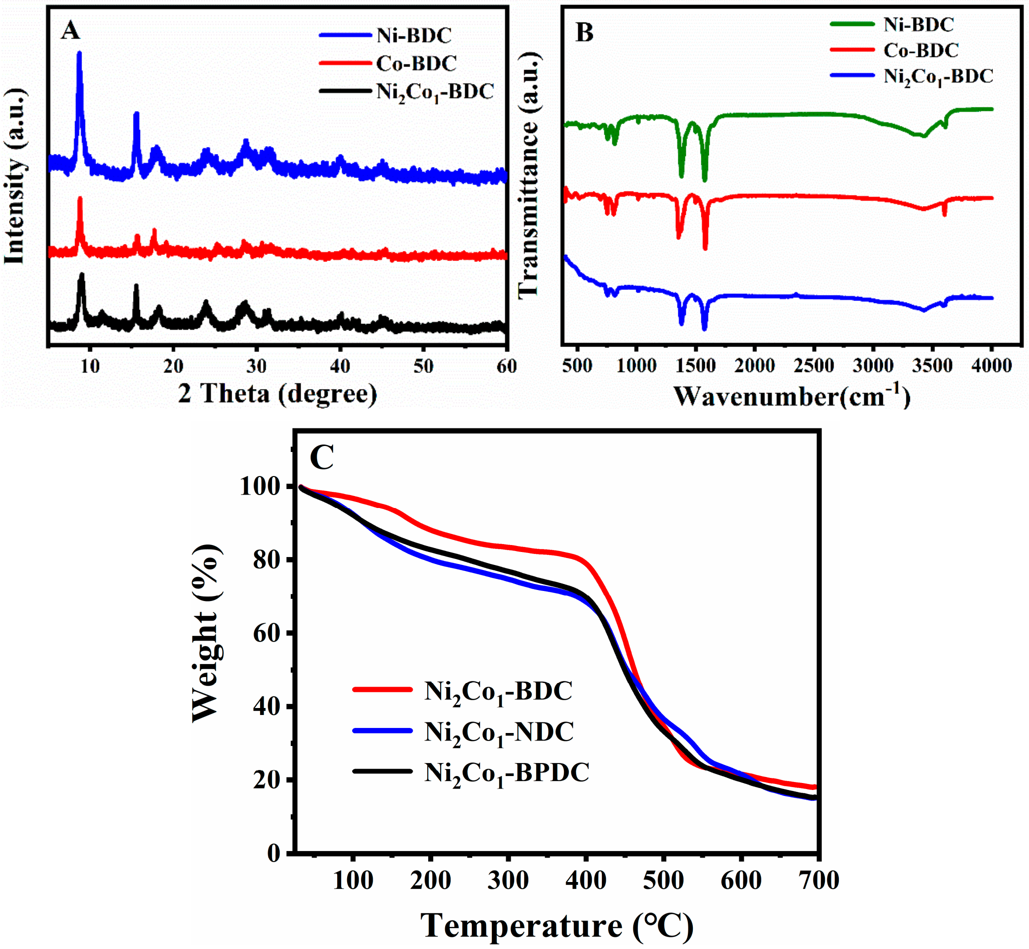

Figure S1. (A) XRD and (B) FTIR spectra of monometallic and bimetallic MOF; (C) Thermal gravimetric analysis (TGA) curve of Ni_2Co_1-L MOFs.

Table S1. The element content of Ni_2Co_1-BDC.

Element	Weight %	Atomic %	Error %
C K	33.88	53.17	8.65
O K	29.88	35.21	9.03
CoK	12.38	3.96	8.76
NiK	23.86	7.66	6.41

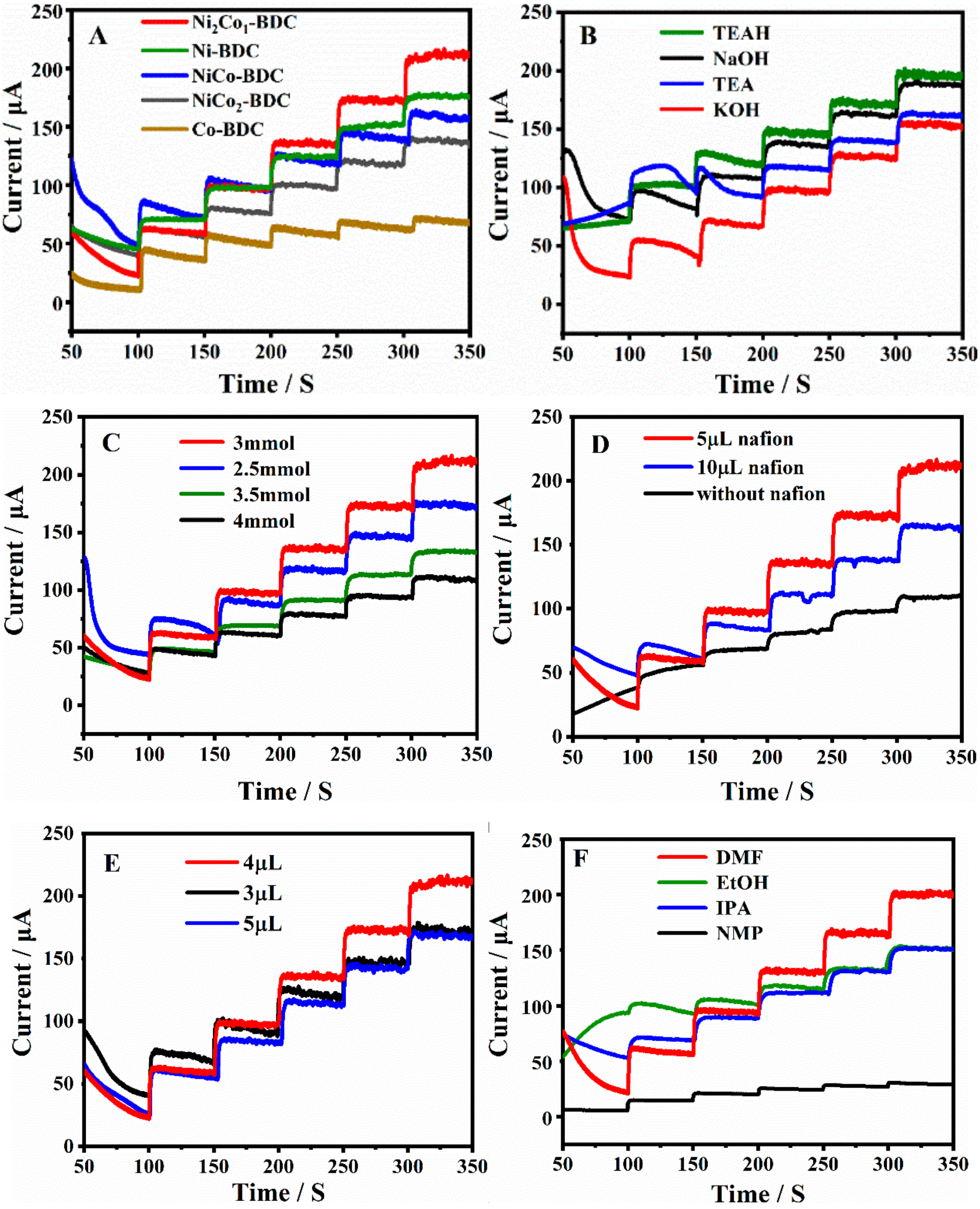

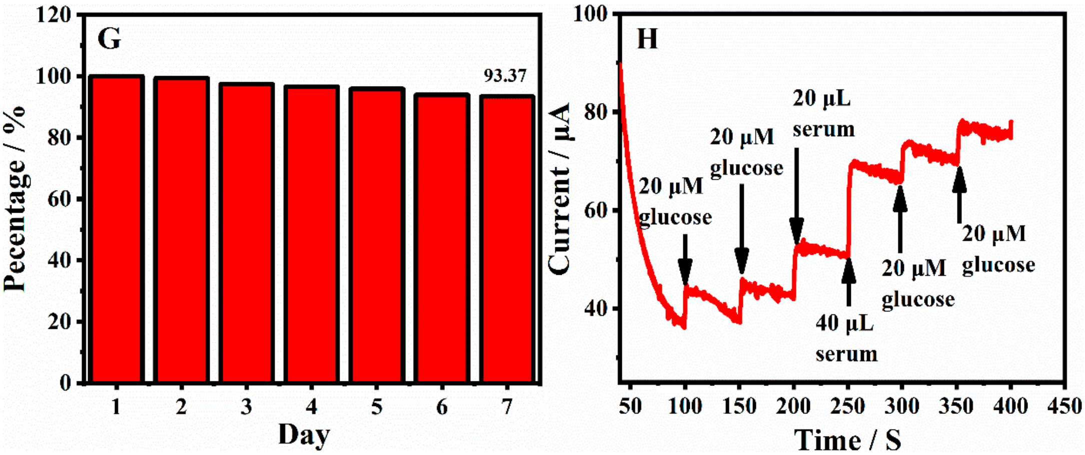

Figure S2. i-t curves of MOFs under different experimental conditions with the continuous injection of 0.1 mM glucose. (A) metal center and scale; (B) types of alkali; (C) amounts of KOH; (D) the volume of Nafion; (E) modified volume; (F) dispersing solvents; (G) stability of Ni_2Co_1-BDC/GCE in 0.1 M NaOH solution containing 0.1 mM Glu; (H) Amperometric response of Ni_2Co_1-BDC/GCE at successive additions of 20 μM glucose, 20 μL serum and 40 μL serum, followed by 20 μM glucose.
